# Detection of Biomarker Clusterin in SERS Immunoassays on Al Foil After Substrate Selection and Assay Optimization with Fluorescently Labeled Antibodies

**DOI:** 10.3390/molecules30193974

**Published:** 2025-10-03

**Authors:** Saule Mergenbayeva, Xeniya Terzapulo, Rostislav Bukasov

**Affiliations:** Department of Chemistry, School of Sciences and Humanities, Nazarbayev University, Astana 010000, Kazakhstan; saule.mergenbayeva@alumni.nu.edu.kz (S.M.); xeniya.terzapulo@nu.edu.kz (X.T.)

**Keywords:** surface-enhanced Raman spectroscopy (SERS), clusterin, aluminum foil, LOD, SERS substrates, SERS immunoassays

## Abstract

Clusterin plays an important role in carcinogenesis and serves as an important diagnostic biomarker of various clinical conditions. This work describes an application of a surface-enhanced Raman scattering (SERS)-based immunoassay using Al foil substrate that has the potential for the detection of clusterin. We first optimized the parameters of the assay using anti-human IgG/human IgG (hIgG) as a model antibody/antigen system using various substrates based on Au film, Si, Al tape and Al foil. Among the tested substrates, Al foil exhibited better performance, when assay of human IgG on Al foil demonstrated a detection limit of 2 pM and a semi logarithmic trend range from 10 pM to 1000 pM. Afterwards, the same SERS immunoassay method was implemented for detection of clusterin and resulted in a good semi-logarithmic calibration line with a high R^2^ value of 0.99, which was obtained in the range from 1 ng/mL to 1000 ng/mL. The low detection limit for clusterin antigen was found to be 3 ng/mL, which is better than most LODs for clusterin reported in the literature and also nearly 4 orders of magnitude lower than possible concentrations of clusterin in human blood. Moreover, the assay requires a relatively low volume of sample (10 μL). Overall, the assay performance demonstrates the significant potential of SERS on Al foil as a low-cost/high-availability substrate for sensing and biosensing, including detection of cancer biomarkers.

## 1. Introduction

A biological marker (biomarker) is a distinctive molecular indicator of a biological/health state that can be evaluated and utilized for early detection of disease. Clusterin is a stress-activated, cell-protecting protein [1]. So far, two sets of different clusterin isoforms have been described: a nuclear isoform (nCLU) with molecular weight (MW) in the range of 50–55 kDa and a secretory isoform composed of two 40 kDa subunits resulting in a heterodimeric glycoprotein complex (sCLU) with MW within the range of 75–80 kDa [2]. It has garnered significant attention since it was recently suggested as a potential biomarker for various diseases ranging from cancer (colorectal cancer [3], bladder cancer [4], breast cancer [5], etc.) to neurodegenerative disorders (e.g., Alzheimer’s disease) [6]. For instance, Pucci et al. [7] found a significant increase in clusterin levels in colon cancer patients. The observed clusterin concentrations in blood were 57.8 ± 19.3 µg/mL (1.445 µM) in the controls and 82.8 ± 26.9 µg/mL (2.07 µM) in patients. Shabayek et al. found that combining angiogenin and clusterin can enhance sensitivity in diagnosing bladder cancer, with a clusterin cutoff value of 15 ng/mg (0.1875 nM) [4].

It also has potential applications in predicting end-stage kidney disease [8] and possibly serving as a cerebrospinal fluid biomarker for human-resistant epilepsy [9]. In the latter case, the concentration of clusterin observed in the drug-resistant group was 49.69 ± 11.11 ng/mL, 251.83 ± 93.16 ng/mL in the drug-responsive group, and 328.10 ± 140.86 ng/mL in controls, respectively.

The disease-prognosis-related studies indicate that the role of clusterin is determined based on changes in its concentration. For example, the concentration of clusterin in human serum ranges from 100 to 300 μg/mL (1.25 pM to 3.75 pM) [10], while the lowest clusterin concentration used in reported studies was higher than ~15 ng/mL (0.1875 pM) [4]. Therefore, technical enhancements are imperative to improve the platform’s limit of detection (LOD) for clusterin, aligning it with the levels typical for most clinically relevant markers, particularly within the low ng/mL and pg/mL range.

So far, a variety of techniques have been investigated to detect clusterin, including bio-sensing [11], single optical fiber-based surface plasmon resonance [12], paper-based lateral flow immunoassay, enzyme-linked immunosorbent assay [13,14], and mass spectrometry [15]. Among the reported studies, Aguilar-Mahecha et al. developed reverse-phase protein microarrays and achieved an LOD of 780 ng/mL (0.021 nM) for clusterin in human plasma using fluorescent-tagged secondary antibodies [16]. Brazaca et al. modified gold nanoparticles with selective antibodies as immunoassay probes. They integrated these probes into paper, producing a millimeter-size colorimetric paper-based device capable of simultaneous analysis of two additional Alzheimer’s disease (AD) biomarkers (fetuin B and clusterin) in human blood samples by achieving an LOD of 0.12 nM by colorimetry and LOD of about 1–3 pM obtained with electrochemical detection, where only three different concentrations and blank were measured and relatively low R^2^ (0.96) and high RSD (26%) were reported. [17]

In the last 20 years, surface-enhanced Raman scattering (SERS) technology has emerged as a highly sensitive, non-invasive, and label-free analytical technique, presenting significant prospects for detecting biological and chemical molecules in trace amounts [6]. Sandwich immunoassays with SERS detection demonstrated high sensitivity, for example, in detection of femtomolar concentrations of prostate specific antigen (PSA) reported by Porter Group [18]. There are multiple advantages of SERS sandwich immunoassays for detection of biomarkers in comparison with fluorescence-based assays [19]. They include, for instance, the ability for simultaneous detection of several biomarkers, known as multiplexing [20]. There are an increasing number of applications of sandwich SERS immunoassays for detection of biomarkers, including detection of cancer biomarkers [21,22,23].

To date, a variety of solid substrates have been extensively tested for SERS detection in biological diagnosis [24,25]. Among them, classical SERS-active substrates are based on noble metals, including gold (Au) and silver (Ag). SERS substrates on Au and Ag possess localized surface plasmon resonances (LSPR) within the visible and near-infrared spectral ranges. Most of the SERS immunoassays are conducted on gold substrates, but since there is significant non-specific protein binding on gold due, for instance, to dative S-Au bonding of sulfur containing amino acids, which are present in practically all protein molecules, there are examples of alternative substrates. Those substrates, reported in the literature recently, include silicon and aluminum, which, in general, have weaker non-specific interactions with proteins and therefore potentially better specificity/selectivity in sandwich immunoassays when compared to those parameters in assays on gold film [26,27]. However, the relatively high cost and susceptibility to corrosion, recrystallization, and biodegradation limit the application of SERS substrates on Au and Ag [28,29].

Recently, aluminum (Al), a universally available metal [30], has attracted worldwide interest due to its sustainable characteristics [31] and lower cost compared to Ag and Au, indicating its potential as a substitute for noble metals. It has proven to be an efficient plasmonic material for applications in the UV-blue light range, particularly in LSPR and enhancing surface fluorescence. Both computational simulations and experimental evidence have demonstrated the effectiveness of aluminum as a material for SERS in the UV spectral range [30]. It has also been reported that aluminum foil purchased from the grocery store is capable of producing SERS substrates with excellent enhancement characteristics [32]. For example, Nguyen et al. [33] developed silver@silica nanocubes on flower-like alumina-coated etched aluminum foil substrate to detect biological systems (creatinine and flavin adenine dinucleotide) typically present in human blood and urine. The prepared sandwich-structured substrate based on alumina-coated etched aluminum foil allowed identification of low concentrations of these biomolecules in artificial urine with LOD values of 0.5 µM and 5 nM for creatinine and flavin adenine dinucleotide, respectively. Treerattrakoon et al. [34] utilized ultra-high vacuum aluminum foil-covered microscope slides for SERS assays to test microvolume sample detection of miR-29a, which is considered as a biomarker for multiple cancer types. They achieved a good linear correlation within the range 0–1000 pM, and the lowest detectable concentration was estimated to be 10 pM. To the best of our knowledge, there has been no study conducted to detect clusterin using a simply prepared SERS-active substrate based on aluminum foil.

The scheme of the SERS immunoassay based on co-adsorption of Raman marker molecules and capture antibody on the surface of gold nanoparticles and physisorption of the same capture antibody on the substrate (gold) was introduced by the Porter group as described in a 1999 publication [35]. In 4 years, the same group reported the application of ERLs/nanotags covered with 5,5-Dithiobis (succinimydil-2-nitrobezoate) or DSNB, which was predicted to serve as a simultaneous Raman reporter and a simultaneous linker of antibody in a highly sensitive detection of prostate specific antigen (PSA) [18]. Numerous applications of this method were reported by multiple research groups, and some of them, along with the method itself, are described in a Tutorial Review published in Chemical society reviews [36]. The same research group that published the above-mentioned review, which had used compounds with N-hydroxysuccinimide (NHS) ester terminal groups (dithiobis (succinimidyl propionate) DSP, DSNB) to covalently link antibodies to gold surface, as they had been doing for over a decade, reported an interesting experimental study in 2014. They studied rates of aminolysis and rates of hydrolysis for one of those compounds (DSP) with IR spectroscopy. They found that under relevant conditions (pH 8.5 and the same concentration of antibody/protein as one used in the immunoassays), the rate constant of hydrolysis was three orders of magnitude higher than the rate constant of aminolysis. In other words, they revealed that a very small fraction of linker molecules may actually covalently link the capture antibody. Therefore, proteins including antibodies would be much more likely to be physisorbed on the substrate and on the surface of a nanotag than to be covalently linked, as had been considered previously [37]. We already demonstrated that immunoassays with SERS readout based on physisorption of the same capture antibody (anti-human IgG) on the surface of the substrate and on the surface of gold nanoparticles, which are partially covered with Raman reporter (4-NBT), can efficiently detect human IgG on two different cost-effective non-noble metal substrates: silicon and Al foil [26,27].

In the present study, Al foil (matte side) was used as a cost-effective substrate in a simultaneous SERS immunoassay for the detection of clusterin, a representative model biomarker. Furthermore, we evaluated and compared the effectiveness of the Al foil substrate with that of the gold (Au) substrate in identifying human IgG (hIgG), serving as the probe molecule for the SERS test. The results illustrate a good detection limit and a strong linear relationship between SERS intensity and the concentrations of probe molecular solutions (clusterin and hIgG).

## 2. Results and Discussion

### 2.1. The Optimization of Assay Parameters

A series of experiments was conducted using fluorescently labeled antibodies, specifically anti-human IgG antibody conjugated to ATTO 647 dye. These experiments aimed to optimize various parameters of the assay across different substrates, including gold film (Au), silicon (Si), and both the matte and gloss sides of aluminum foil (Al_matte and Al_gloss), as well as aluminum tape (Al tape). Figure S1 in the Supplementary Materials illustrates the variations in fluorescence intensity of the ATTO 647 dye, detected at approximately 663 nm, depicted for different exposure times (2, 4, and 8 h) on the aforementioned substrates. It is evident from the figures that the fluorescence signal increases proportionally with the duration of exposure for all the substrates under investigation. This trend suggests that longer exposure times lead to enhanced detection sensitivity across the various substrate types, thereby optimizing the assay conditions for reliable results.

Furthermore, the observed significant increase in fluorescence intensity with extended exposure times underlines the importance of optimizing assay parameters to maximize signal sensitivity across diverse substrate compositions. The comparable signals observed on aluminum foil, whether matte or gloss, and gold substrates suggest a consistent performance of the assay across these materials, potentially due to their favorable surface properties for antibody binding [38] and dye detection [39]. Conversely, the lower signal detected on Si substrates may indicate challenges in achieving effective antibody binding or dye interaction, possibly due to differences in surface chemistry or substrate morphology. These substrate-dependent variations highlight the need for tailored optimization strategies to ensure robust assay performance across different material platforms. Additionally, the thorough approach employed in conducting the measurements, including the use of a 633 nm laser excitation source and x10 objective lens, and the acquisition of data from multiple spots on duplicate samples, ensures the reliability and reproducibility of the experimental results, thereby strengthening the validity of the conclusions drawn from this comprehensive investigation.

**Figure 1:**
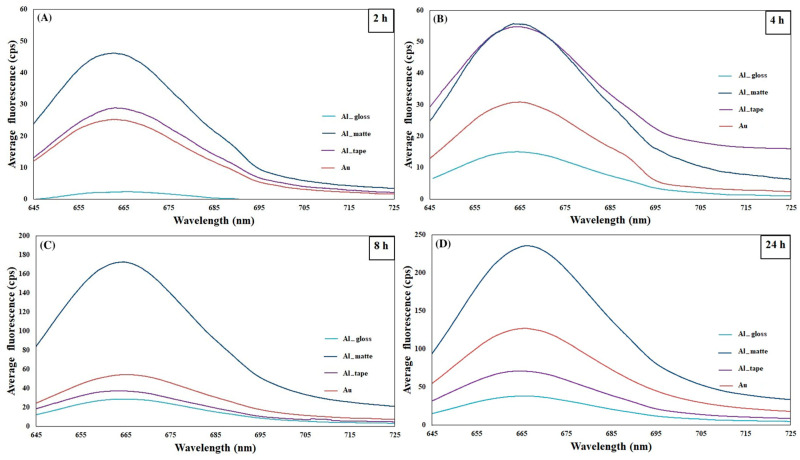
Fluorescence emission spectra for Al_matte, Al_gloss, Al_tape and Au substrates obtained with a 633 nm laser with the exposure time of (**A**) 2 h, (**B**) 4 h, (**C**) 8 h and (**D**) 24 h. Signal obtained from 4 µg/mL ATTO647 labeled antibody.

To further investigate the substrate performance, an additional experiment was undertaken using human IgG as the capture antibody and ATTO 647-labeled anti-human IgG as the antigen. These experiments were specifically designed to assess the efficacy of the substrates, focusing on Au and Al substrates, including aluminum tape and both matte and gloss surfaces of aluminum foil. By varying the duration of exposure to the antigen, a comprehensive understanding of substrate suitability and performance dynamics was obtained. As depicted in Figure 1, the results demonstrate a pronounced trend of increasing fluorescence intensity with prolonged durations of exposure to the antigen (analyte). Notably, the fluorescence signal on aluminum tape surpassed that of gold substrates for 2 and 4 h of exposure; extending the exposure time to 24 h resulted in a more substantial enhancement in signal intensity on Au substrates. Among the aluminum substrates investigated (tape, matte, and gloss surfaces), aluminum foil, particularly Al_matte, emerged as the best performer across all tested durations. Notably, the performance of Al_matte surpassed that of gold by nearly 1.8 times, emphasizing its superior efficacy as a substrate for the assay. Moreover, the 24 h experiment showed that the signal improvement followed the order of Al_matte > Au > Al tape > Al_gloss, with Al_matte exhibiting the most substantial improvement.

During the optimization experiments previously described, it became evident that aluminum foil substrates, including both the matte and gloss sides, outperformed the gold (Au) substrate. It was observed that reducing the antigen concentration from 40 µg/mL (Figure S1) to 4 µg/mL (as depicted in Figure 1) had an impact on the performance of the Al_gloss substrate. Conversely, the performance of the Al_matte substrate remained relatively unaffected by this alteration in antigen concentration. Given the pronounced stability in performance exhibited by the Al_matte substrate under varying experimental conditions, it was deemed judicious to select it for further SERS investigations aimed at detecting human IgG (hIgG) and clusterin. These findings highlight the importance of carefully selecting substrates with robust and consistent performance characteristics, particularly in assays that have high sensitivity and good reliability requirements.

### 2.2. The Detection of hIgG

**Figure 2:**
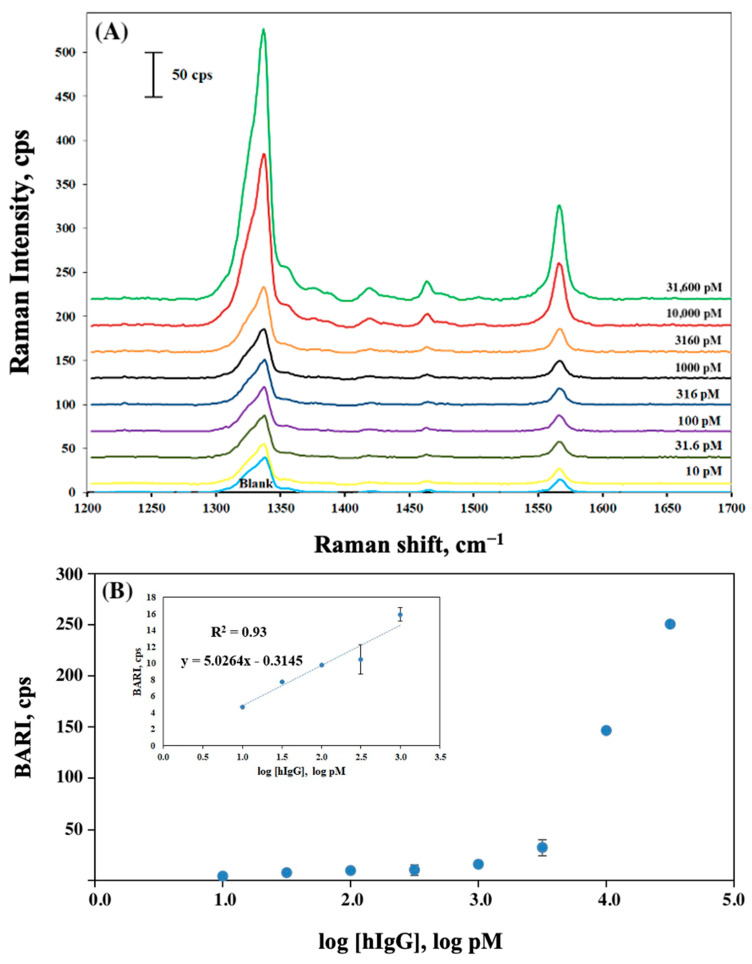
(**A**) Concentration-dependent SERS spectra for hIgG (10 pM to 31,600 pM), shown with background offset for Al_matte substrate. (**B**) Calibration plots for blank-adjusted Raman intensities (BARI) vs. the different concentrations of hIgG on Al_matte substrate. Inserted calibration plot is used for human IgG LOD calculation on Al_matte.

The analytical performance of Al_matte substrate was further tested to detect various concentrations (ranging from 10 pM to 31,600 pM) of hIgG, used as a model biomarker. SERS measurements were performed for eight different concentrations, with each sample prepared in duplicate to ensure robustness and reproducibility of the results. As expected, the Raman signal intensities (Table S1) exhibited a noticeable increase with the increasing hIgG concentrations (Figure 2A), and the corresponding function of blank adjusted Raman peak intensity (BARI) as a function of analyte/hIgG concentration is presented in Figure 2B. These results demonstrate potential usability of the Al_matte substrate as a versatile platform for sensitive, quantitative biomarker detection.

To establish calibration curves, a series of antigen concentrations (ranging from 10 to 1000 pM) was used. The LOD for the assay using the Al_matte substrate was estimated using the 3σ rule (where σ represents the standard deviation of the blank) and was found to be 2 pM. The calculated LOD value for hIgG in a sandwich SERS immunoassay on Al_matte, determined using the most conservative estimate, highlights the exceptional sensitivity enabled by the Al_matte substrate, which is comparable to or even lower than those reported previously by Kunushpayeva et al. [27]. They obtained LODs of 25 pM and 34 pM on Si and Au substrates, respectively, indicating that the LOD obtained in this study using the Al_matte substrate is 12.5 and 17 times lower than those for the Si and Au substrates.

Furthermore, the same data were utilized for LOD calculation using the three-parameter logistic model (3PL) (Figure S2).

### 2.3. The Detection of Clusterin

**Figure 3:**
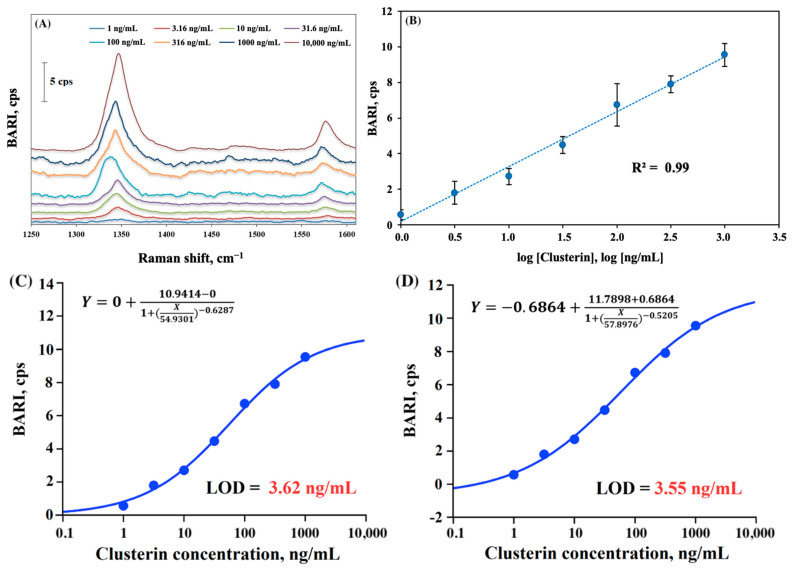
(**A**) SERS spectra and (**B**) calibration plot in SERS immunoassay of clusterin at various concentrations of target clusterin on Al foil matte substrate, obtained with a 633 nm excitation laser. (**C**) Three- and (**D**) four-parameter logistic nonlinear regression analysis calibration plots for detection of clusterin in SERS sandwich immunoassay on Al foil matte substrate.

Next we applied Al_matte substrate to detect clusterin as a target biomarker. Table 1 shows the numerical outcomes of this assay. Figure 3 shows eight blank-adjusted Raman spectra (Figure 3A) for eight different concentrations of biomarker, ranging from 1 to 10,000 ng/mL. Similarly to the experiment implemented for the detection of hIgG, a clear trend of the Raman intensity’s growth with an increase in the concentration of clusterin was observed. Notably, the presence of clusterin at a concentration of 10,000 ng/mL resulted in the highest SERS intensity. Figure 3B represents a semilogarithmic calibration curve for the SERS sandwich immunoassay of clusterin. The Raman measurements revealed a significant semilogarithmic increase in SERS intensity (R^2^ = 0.99 for BARI vs. log[clusterin]) with the rise in antigen concentration ranging from 1 to 1000 ng/mL. LOD for the target clusterin was calculated from the trendline of signal versus logarithm of clusterin concentration, yielding a value of 59.5 pM (corresponding to ~3.03 ng/mL).

In addition, three- and four-parameter logistic models (3PL and 4PL) were employed for LOD calculation (Figure 3C,D), as this approach is commonly used for calibration in bio-detection methods such as ELISA [40]. Consistent with the LOD value for clusterin obtained using linear logarithmic calibration, the LOD values calculated using the 3PL and 4PL models were determined to be 71.1 pM (3.62 ng/mL) and 69.7 pM (3.55 ng/mL), respectively. Notably, these obtained LOD values were estimated within the same range of clusterin concentrations, using seven data points (as summarized in Table 1), as those obtained from the logarithmic calibration approach.

The measurements performed after 2 months of sample storage in a dark place, revealed LOD values of 48 pM (2.46 ng/mL), 43 pM (2.2 ng/mL), and 50 pM (2.5 ng/mL) for linear, 3PL, and 4PL calibrations, respectively. These values, presented in Table S3, were approximately 1.2–1.6 times lower/better than those obtained from measurements in the first week after the assay, suggesting a potential improvement or stabilization of the assay performance over time with storage. The calibration plots for the 3PL and 4PL calibrations in the second measurement, conducted after the extended storage period, are depicted in Figure S3A,B. Despite the decrease in LOD values, the intensity for the second measurement was reduced, indicating that the re-measured data showed lower signal strength. Two-dimensional diffusion/movement of some nanotags on a substrate surface may result in more uniform distribution of nanotags on the surface due to this motion. That can explain this improvement in sensitivity, driven mostly by a decrease in standard deviation of the blank from 0.56 to 0.23 cps of the Raman signal. The decrease in SD of the blank is likely to come from more uniform distribution of nanotags on the surface due to this 2D motion and dissembling of some dimers, trimers, and oligomers due to this lateral diffusion of nanoparticles, which would result in a more reproducible signal of the blank, where nanotags in the absence of antigens are only nonspecifically bound to the surface of the substrate.

It is important to note that a suboptimal amount of anti-clusterin antibody was used in this SERS immunoassay (about 70–75% in comparison to the amount used in the hIgG assay), since the capture antibody is relatively expensive. There is still room for improvement in the assay, particularly in increasing the antibody concentration, which is likely to enhance the sensitivity and accuracy of clusterin detection.

**Figure 4:**
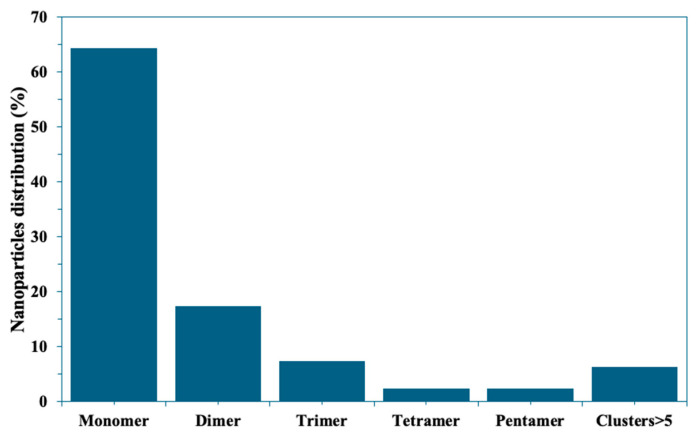
Distribution of aggregates based on the number of Au NPs.

Figure S5 demonstrates an SEM image showing commercial 50 nm AuNPs with a scale bar of 100 nm. The SEM analysis of the applied commercial AuNPs in the SERS immunoassay reveals their spherical morphology, with diameters averaging approximately 50 nm. A statistical analysis of SEM images (Figure 4), based on the examination of over 339 nanostructures from five different regions, reveals that the majority are isolated Au NPs (64.3%), followed by Au NP dimers (17.4%). Additionally, triangular trimers accounted for 7.4% of the nanostructures, with tetramers and pentamers each constituting 2.4%, and oligomers (>5) making up 6.2%.

The analysis of nanostructure distribution, particularly the formation of dimers and trimers, is crucial for understanding the enhanced Raman signal observed in the SERS immunoassay [41]. Dimers and trimers of Au nanoparticles are known to exhibit strong LSPR coupling effects [42], which significantly enhance the electromagnetic field between the closely spaced nanoparticles. This phenomenon is referred to as the ‘hot spot effect’, which plays a critical role in amplifying the Raman signal [43]. The observed proportions of dimers (17.4%) and trimers (7.4%) suggest that these configurations contribute significantly to the SERS signal enhancement. In particular, dimers often generate the most intense hot spots due to their well-defined interparticle gaps, while trimers can provide additional coupling effects through multi-particle interactions.

Not only can SEM observe nanotag morphology, but analysis of SEM maps, like those shown in the Figure S6, can be used to estimate the number of nanoparticles per unit area, as illustrated in the Figure S4 for various clusterin concentration. This observation highlights a robust correlation between the number of nanoparticles per µm^2^ and clusterin concentration, with a significant R^2^ value of 0.989. The quantified numbers of nanoparticles per µm^2^ in relation to clusterin concentration are tabulated in Table S4, and the trend is shown in in the Figure S4, while the similar R^2^ (0.99) was calculated on a Raman calibration plot for the same clusterin assay.

AuNPs and SERS nanotag samples were tested by UV-visible spectroscopy to assess the successful functionalization of AuNPs with clusterin antibody. Figure S7 shows that PBS-capped AuNPs display characteristic surface plasmon resonance (SPR) at 538 nm [44]. The characteristic peak of AuNPs slightly shifted after 4-NBT and clusterin antibody modification, by Δλ of about 4 or 5 nm, indicating that the 4-NBT and antibody modified the surface of AuNPs [45].

Overall, the LODs obtained by SERS sandwich immunoassays were relatively well reproduced by three different types of calibrations (semilogarithmic, 3-P and 4-P fits) and the LOD values were quite low in comparison to most LODs for clusterin reported in the literature.

## 3. Materials and Methods

### 3.1. Materials

Commercial microscope glass slides (Corning Incorporated, Corning, NY, USA) were coated with aluminum tape and foil to prepare substrates. Si wafers, commercial microscope slides coated with gold films of 100 nm thickness (99.9% purity), over Cr (thickness = 2–3 nm) layer, were purchased from EFM Co, Carson, CA, USA. Native human IgG protein (Abcam, AB205806, Cambridge, Cambridgeshire, UK), anti-human IgG antibody produced from goat (H + L) coupled to ATTO647N (Hypermol), anti-human IgG antibody produced from goat (CSB_PA793692), and human clusterin protein (MW: 50.9 kDa) were obtained from ProSci Incorporated (Poway, CA, USA), while clusterin recombinant monoclonal antibody derived from human clusterin (CSB-RA449834A0HU) was purchased from Cusabio Technology LLC (Houston, TX, USA). SuperBlock™ blocking buffer in TBS (SBB in TBS) and Borate Buffer (BB, pH = 8.5) were purchased from Thermo Scientific. A Stabilized suspension of gold nanoparticles (753645-25 mL, in 0.1 mM PBS) with the average diameter of 50 nm, Bovine Serum Albumin (BSA, A7030-10 g), Twin 20 detergent (P1379-25 mL), 4-nitrobenzenethiol (4-NBT), and Phosphate Buffered Saline (PBS, P4417) were obtained from Sigma Aldrich (Gillingham, UK). Ultra-pure water (UPW) generated from a Millipore purification system was used for all aqueous solution preparations.

### 3.2. Measurements

SERS measurements were implemented using a confocal Raman spectrometer (Horiba, LabRam HR evolution, Kyoto, Japan) equipped with a thermoelectrically cooled charge-coupled device (CCD) detector, 633 nm Helium-Neon laser source with power of approximately 5 mW, ×10 objective, 600 lines/mm grating, and a neutral-density filter ratio of 100% for all substrates. The measurements for ATTO647 dye were implemented in the spectral range of 645–725 nm, while for hIgG and clusterin, they were implemented in the spectral range of 1200–1700 cm^−1^. The acquisition time was set at 2 s. SEM images of 50 nm AuNPs were obtained using a scanning electron microscope Zeiss Crossbeam540 (Zeiss, Oberkochen, Germany). UV-vis measurements of AuNPs and SERS nanotags were implemented using a microtiter plate reader (Synergy H1 Multi Mode Reader, BioTek, Winooski, VT, USA).

### 3.3. Preparation of SERS-Active Substrates

First, the glass microscope slides, Au film and Si wafer were cut into pieces with a size of 1.3 × 1.3 cm (0.5 × 0.5 in.). Then, to prepare Al substrates, small glass squares were coated with Al foil (matte and gloss sides) and tape, followed by cleaning with ethanol and UPW. In the next step, a piece of Parafilm with an approximate 5–6 mm diameter hole in the center was gently pressed and then thermally sealed onto the surface of Au, Si, and the glass squares covered with Al foil and tape. However, for the immunoassay with clusterin, the diameter of the hole was 3 mm. The latter step allows for confining the droplet of aqueous solution to the center, attributed to the hydrophobic nature of parafilm. Then, all the prepared substrates were rinsed with ethanol and water. Afterward, the substrates were placed into a mini-wet chamber, where they were surrounded by water droplets and covered with inverted Petri dishes. The reason for conducting the assay within these mini-wet chambers was to prevent solution droplets from drying during the procedure.

### 3.4. Assay Protocol

#### 3.4.1. Assay Protocol of Optimization Experiments

**Figure 5:**
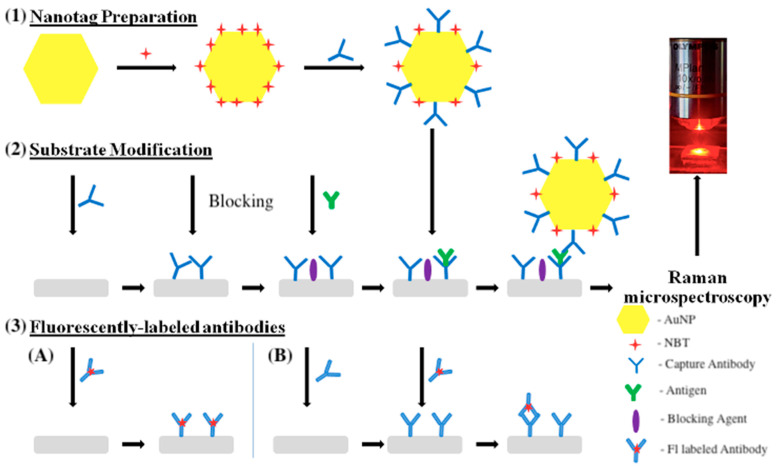
Schematic illustration of SERS immunoassay using fluorescently labeled antibodies.

First, 30 μL of a PBS solution containing antigen at a concentration of 40 μg/mL was dropped onto the substrates (5–6 mm in diameter) and incubated for 4 h in a moist chamber at room temperature (RT). Afterward, the substrates were washed twice with PBS solution. The samples were then blocked using SBB in TBS for 4 h at RT, followed by washing twice with PBS solution. Then, anti-human IgG antibody conjugated to ATTO 647 dye (40 μg/mL or 4 μg/mL) was diluted in PBS and incubated with samples for 2 h, 4 h, 8 h and 24 h. Samples were then washed with PBS to remove the unbound antibodies. The assay stages are represented in Figure 5.

#### 3.4.2. Assay Protocol for hIgG

The assay procedure for hIgG detection was adopted from Kunushpayeva et al. [27] with some changes, including decreasing the volume for antibody and antigen solutions from 25–30 µL to 10 µL, which was likely to decrease the diffusion time of antigen/antibody from bulk to the substrate, where antigen-antibody binding that occurs is likely to increase the efficiency of the assay.

#### 3.4.3. Assay Protocol for Clusterin

The assay procedure for clusterin detection was similar to that applied for hIgG. However, due to the limited availability of chemicals and materials, optimizations were performed. The assay involves four stages. After the initial three stages, the substrates were rinsed using PBS solution with a pH of 7.40.

First, 10 μL of a 40 μg/mL clusterin recombinant monoclonal antibody in PBS solution was delivered to each substrate and then all substrates were put in wet chambers for 4 h. After rinsing each substrate, 10 µL of a blocking buffer was added on each substrate and left for 3 h. Subsequently, 10 µL of clusterin in PBS solution was applied to the rinsed substrates for 4 h. In the last stage, 10 µL of Extrinsic Raman Labels (ERLs) suspension was added to the surface of each substrate and left for 8 h. Finally, the substrates were rinsed with 1 mL of 0.1% Tween 20 in PBS.

To form ERLs, 10 µL of a 1 mM solution of Raman reporter (4-NBT) was combined with 1.0 mL of 50 nm AuNPs in PBS and 50 µL of 50 mM BB with a pH of 8.5 in a microcentrifuge tube. The mixture was shaken for 2 h. Then, the suspension was subjected to centrifugation at 9000 rpm for 5 min, followed by discarding the excess 4-NBT from the supernatant. The AuNPs were resuspended in 600 µL of 2 mM BB, and 400 µL of 25 µg/mL of the binding capture antibody (clusterin antibody) was introduced to allow the antibody to adsorb onto the surface of AuNPs, resulting in the formation of the ERLs. The suspension was stirred for 6 h. Then, 120 µL of a 10% BSA solution was added to the tube, and the suspension was shaken for an additional 5 h. The ERL suspension was centrifuged for 8 min at 9000 rpm. The supernatant was decanted, and the AuNPs were resuspended in 1 mL of 1% BSA solution in 2 mM BB. This centrifugation/resuspension process was repeated twice more, with the final suspension in 270 µL 1% BSA solution in 2 mM BB and 30 µL of 10% solution of NaCl to mimic physiological ionic strength close to that of human blood.

## 4. Conclusions

In this study, the application of a surface-enhanced Raman scattering (SERS)-based immunoassay using aluminum foil substrate for the detection of clusterin was investigated. Through the selection of the substrate and optimization of SERS-active immunoassay parameters using anti-human IgG antibody conjugated to ATTO 647 dye, highly sensitive detection of clusterin has been achieved. Our results revealed that the aluminum foil substrate, particularly the matte side, outperformed other SERS-active substrates such as gold film, silicon, and aluminum tape for the detection of hIgG, showcasing its superior SERS activity.

The detection limit for hIgG was estimated to be 2 pM over a concentration range of 10 to 1000 pM using the aluminum foil substrate. Then, we applied the SERS assay with optimized conditions for biomarker clusterin detection. Notably, we observed a linear correlation between the logarithm of clusterin antigen concentration and SERS intensity responses within the range of 1 to 1000 ng/mL (R^2^ = 0.99), underscoring the assay’s reliability and quantitative capabilities.

Furthermore, the detection limit for clusterin detection was determined to be 3.0 ng/mL (60 pM) in a sample volume of 10 μL, which is the equivalent of detecting as little as 30 pg or 0.6 femtomoles or about 10 million molecules of the biomarker. Such a low LOD for clusterin demonstrates the assay’s sensitivity and potential for practical applications in biomarker detection and disease diagnosis. These results contribute to the growing body of knowledge in the field of SERS-based immunoassays and pave the way for future advancements in sensitive and reliable detection methods for clinically relevant biomolecules.

## Figures and Tables

**Table 1:** Raman peak intensity versus clusterin concentration.

Concentration, ng/mL	BARI, cps
1	0.57
3.16	1.8
10	2.71
31.6	4.47
100	6.73
316	7.91
1000	9.55
St. Dev for blank	0.55927
3xSt. Dev.	1.67781
LOD, ng/mL	**3.03**
LOD, pM	**60**

## Data Availability

Data are contained within the article and Supplementary Materials.

## Supplementary Materials

The following supporting information can be downloaded at: https://www.mdpi.com/article/10.3390/molecules30193974/s1, Those materials Include: Figure S1: Fluorescence emission spectra for all substrates obtained with: (A) 2h, (B) 4h and (C) 8h exposure times; Table S1: Raman peak intensity versus hIgG concentration used for 3-P fit calibration; Figure S2: Three-parameter logistic nonlinear regression analysis calibration plot of hIgG using Al_matte substrate; Table S2: Raman peak intensity versus clusterin concentration obtained after a 2-month interval; Figure S3: 3P and 4P Nonlinear calibration plots; Table S3: Results of LOD calculations for Cclusterin using the data obtained after a 2-month interval; Table S4: Measured ERL surface density as a function of the clusterin concetration; Figure S4: The plot of the number of nanoparticles per µm2 versus clusterin concentration; Figure S5 SEM Images of high magnification; Figure S6: Representative SEM images of low magnification (×10,000); Figure S7: UV-Visible spectra of AuNPs and SERS nanotag; Figure S8: Structural formula of 4-Nitrobenzenethiol (NBT).
