# Supplementary material for: Detection of Biomarker Clusterin in SERS Immunoassays on Al Foil After Substrate Selection and Assay Optimization with Fluorescently Labeled Antibodies
Source: Molecules. 2025 Oct 3;30(19):3974. doi: 10.3390/molecules30193974 (PMC12525947; doi:10.3390/molecules30193974)
Supplement: Supplementary file 1 [file molecules-30-03974-s001.zip › molecules-3848729-supplementary.pdf]

Supplementary Information

Detection of Biomarker Clusterin in SERS Immunoassays on Al foil After Substrate Selection with Fluorescently Labeled Antibodies

Saule Mergenbayeva, Xeniya Terzapulo and Rostislav Bukasov *

Department of Chemistry, School of Sciences and Humanities, Nazarbayev University, Astana 010000, Kazakhstan; saule.mergenbayeva@nu.edu.kz (S.M.); xeniya.terzapulo@nu.edu.kz (X.T.); rostislav.bukasov@nu.edu.kz (R.B.)

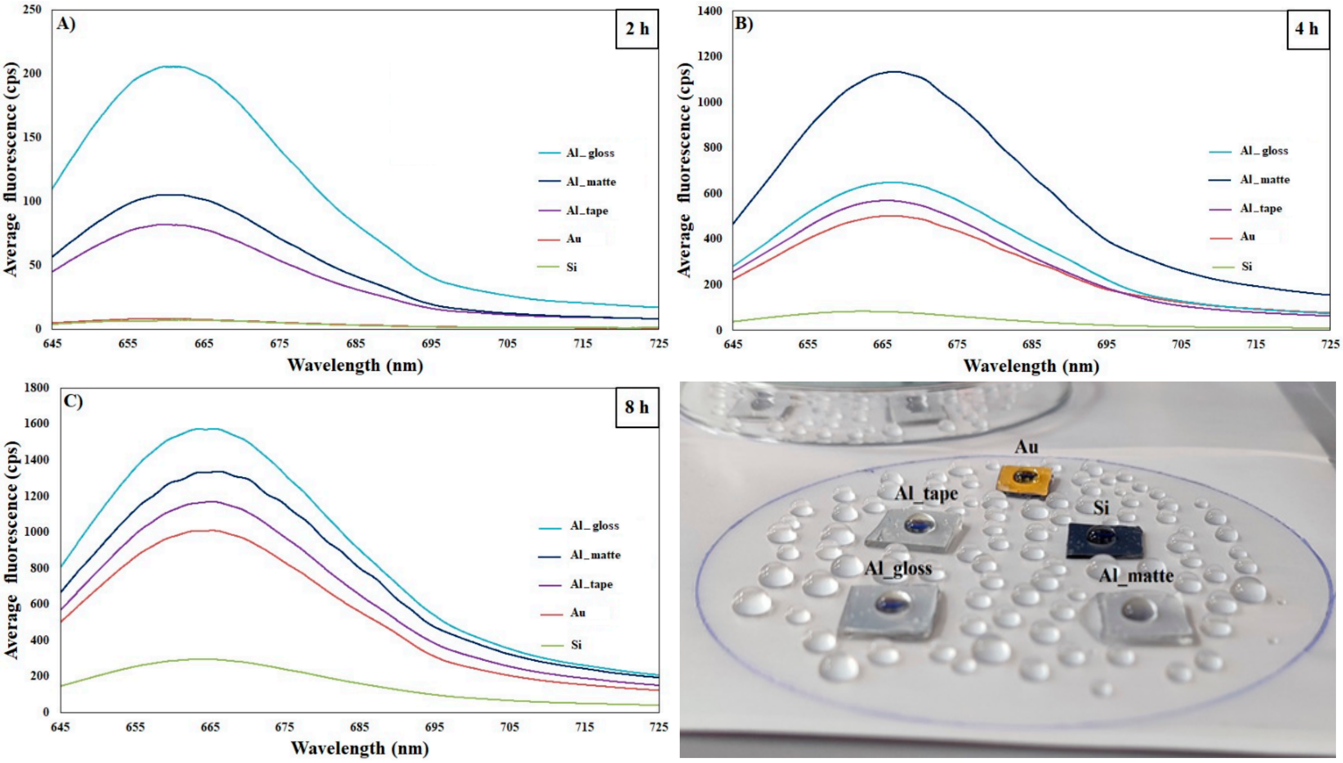

Figure S1. Fluorescence emission spectra for all substrates obtained with: A) 2h, B) 4h and C) 8h exposure times. Signal obtained from ATTO647-labeled antigen (40 µg/mL). D) Pictures of addresses on Au, Si, Al tape, Al_matte and Al_gloss substrates (bottom part of a wet chamber).

Table S1. Raman peak intensity versus hIgG concentration used for 3-P fit calibration.This data is used the plotting three-parameter logistic nonlinear regression analysis calibration plot.

Concentration, pM	Blank Adjusted Raman Signal
10	4.72
31.6	7.78
100	9.78
316	10.47
1000	15.94
3160	32.19
10000	146.65
31600	250.7
St. Dev for blank	0.396
3*St. Dev.	1.188

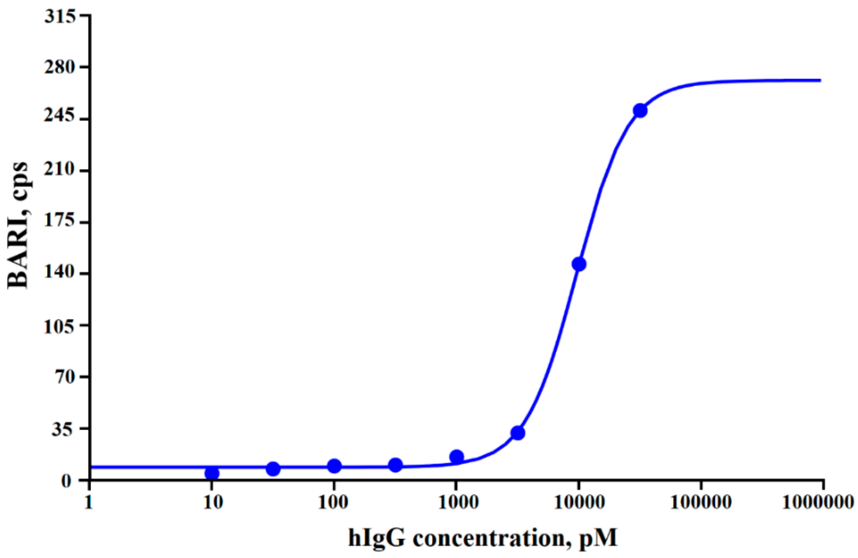

Figure S2. Three-parameter logistic nonlinear regression analysis calibration plot of hIgG using Al_matte substrate.

Eq.1 that was used for 3PL graph:

$$y = \frac{maximum}{1+\left(\frac{x}{x_{50}}\right)^{Hill\,coefficient}} = \frac{286.6655}{1+\left(\frac{x}{9845.8055}\right)^{-1.6756}} \quad (1)$$

Table S2. Raman peak intensity versus clusterin concentration obtained after a 2-month interval.

Concentration, ng/mL	Blank Adjusted Raman Signal
1	0.1049693227
3.16	0.7667700821
10	1.697807459
31.6	2.363753787
100	2.936129562
316	3.605196387
1000	4.503992226
St. Dev for blank	0.23
3*St. Dev.	0.69

The following data was used in plotting three- and four-parameter logistic curves (3PL and 4PL). These regression models were built using 3PL and 4PL curve calculators.[1].

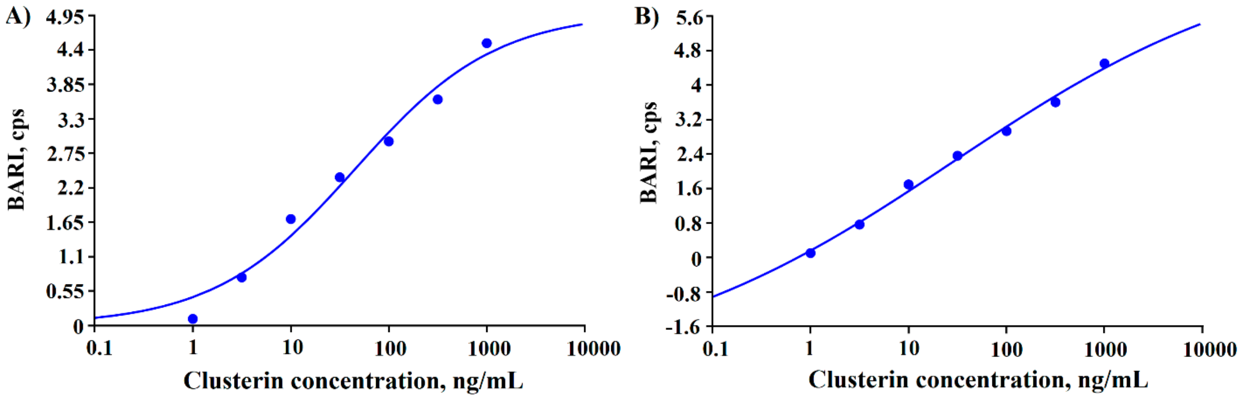

Figure S3. 3P and 4P Nonlinear calibration plots. A) Three- and B) four-parameter logistic nonlinear regression analysis calibration plots of clusterin using Al_matte substrate after a 2-month interval.

Equations used for the 3PL (Eq. 2) and (Eq. 3) graphs:

$$y = \frac{maximum}{1+\left(\frac{x}{x_{50}}\right)^{Hill\,coefficient}} = \frac{4.9945}{1+(\frac{x}{45.4049})^{-0.6046}} \quad (2)$$

$$y = minimum + \frac{maximum-minimum}{1+\left(\frac{x}{x_{50}}\right)^{Hill\,coefficient}} = -2.7019 + \frac{7.1498+2.7019}{1+(\frac{x}{28.505})^{-0.2662}} \quad (3)$$

From the aforementioned Eq. 2 and Eq. 3, the following parameters can be outlined and used for LOD calculations:

$$LOD = \left(\frac{maximum-minimum}{st.dev.for\ blank-minimum} - 1\right)^{\frac{1}{Hill\,Coefficint}} * x_{50} \quad (4)$$

Table S3. Results of LOD calculations for Cclusterin using the data obtained after a 2-month interval.

	3PL	4PL	Using logC
LOD, ng/mL	2.2	2.5	2.46
LOD, pM	43.2	49.8	48.3

The LOD values for this measurement, obtained using different methods (3PL regression, 4PL regression equations, and traditional method), range from 2.2 ng/mL to 2.5 ng/mL (43.2-49.8 pM). These results demonstrate the reproducibility of the obtained LOD values.

Table S4. Measured ERL surface density as a function of the clusterin concetration.

Clusterin concentration (ng/mL)	Measured particle density (ERL/μm²)
Blank	0.041
1	0.048
3.16	0.052
10	0.090
31.6	0.161

The quntitative SEM meassurements with calculation of NP surface density was done about 8 months after immunaossay Raman meassurements, 7.5 months after and agglomeration/association of nanoparticles may increase in this period of time due to 2D difusion/ migration of nannoparticles on the surface. Hovewer total number of NPs and surface density would NOT be affected in this process.

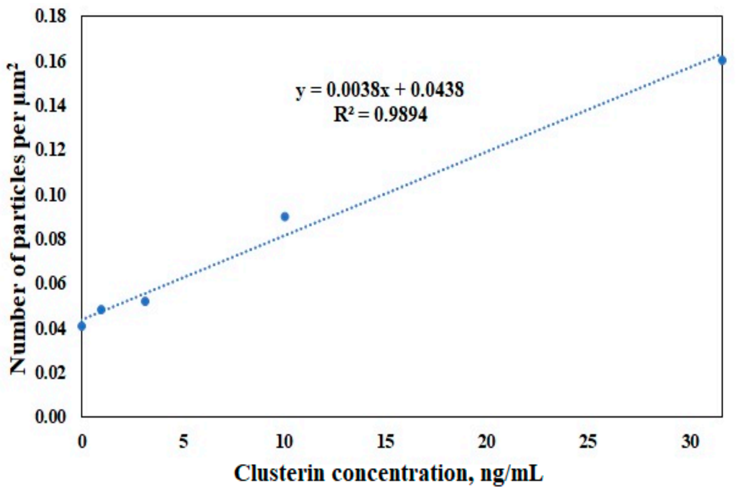

Figure S4. The plot of the number of nanoparticles per μm² versus clusterin concentration.

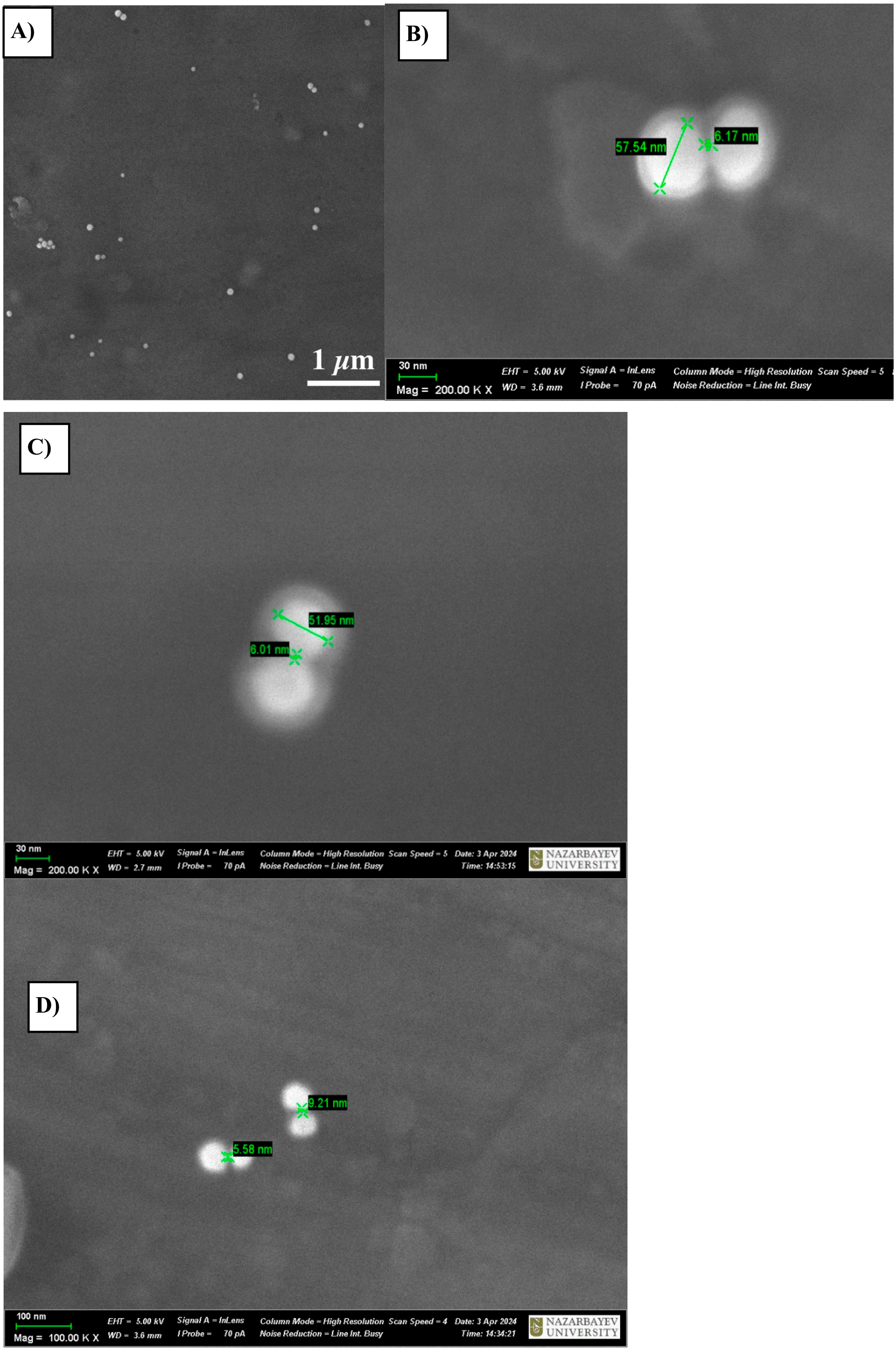

Figure S5. SEM Images of high magnification. SEM images of 50 nm diamter AuNPs as nanotags from human IgG assay. Interparticle diameters and interparticle gaps are meassured and shown on Figure B), C) D) Nonaoparticle gaps meassured in 6 to 9 nm range (with significant uncertainty since it is clode to limit of resolution)) meet our expectations for gap distance, since minimum capture antibody layer thickness is expected to be around 4.5-5 nm.

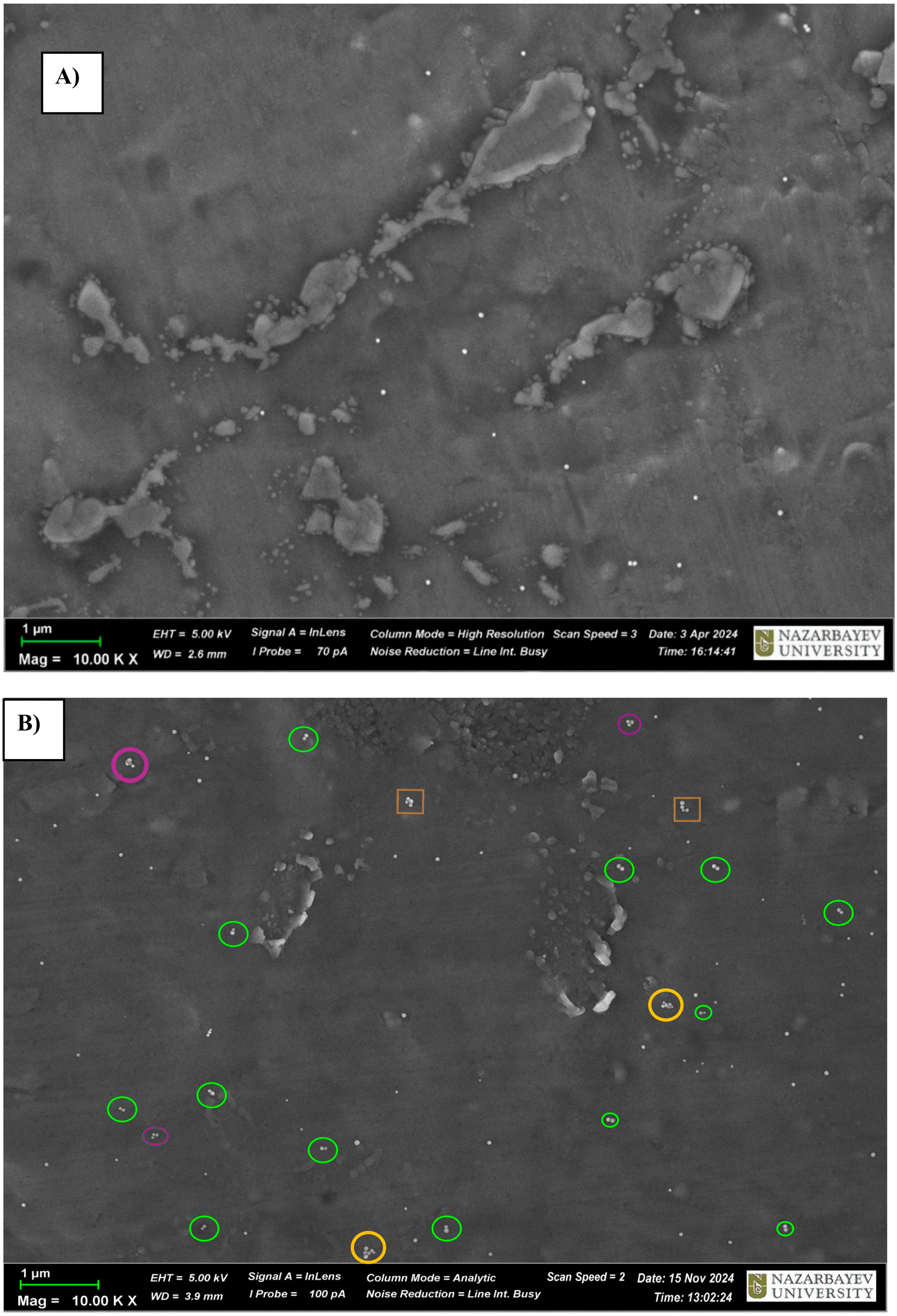

Figure S6. Representative SEM images of low magnification (x10000).

Images taken for samples from two described in the article immunoassays: A) assay of human IgG, B) Assay of clusterin. There is ubiquitous salt from PBS buffer and sometimes bacteria visible on those SEM images as well.

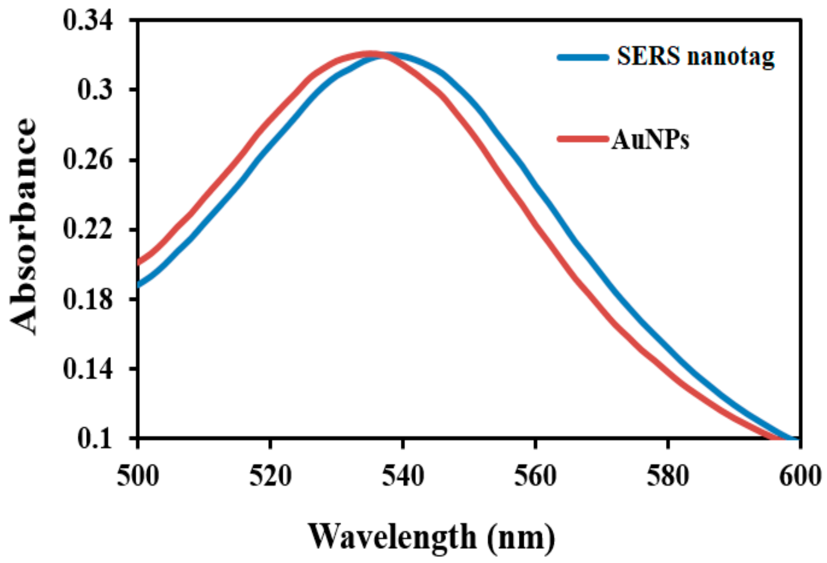

Figure S7. UV-Visible spectra of AuNPs and SERS nanotag.

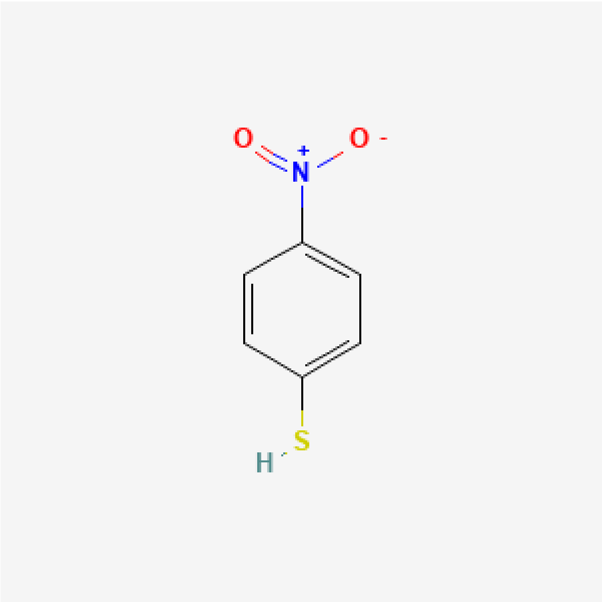

Figure S8. Structural formula of 4-Nitrobenzenethiol (NBT).

(1) Inc, A. A. T. B. *Four Parameter Logistic (4PL) Curve Calculator*. 2024. https://www.aatbio.com/tools/four-parameter-logistic-4pl-curve-regression-online-calculator (accessed.
